# Novel HIV-1 Recombinant Identified in a Foreign Heterosexual Resident in Japan: Relatedness to Recently Reported CRF69_01B, Detected Primarily among Japanese Men Who Have Sex with Men

**DOI:** 10.1128/genomeA.00196-15

**Published:** 2015-05-28

**Authors:** Shigeru Kusagawa, Yuko Yokota, Masayoshi Negishi, Makiko Kondo, Tetsuro Matano, Shingo Kato, Yutaka Takebe

**Affiliations:** a AIDS Research Center, National Institute of Infectious Diseases, Tokyo, Japan; b Negishi Clinic, Tokyo, Japan; c Division of Microbiology, Kanagawa Prefectural Institute of Public Health, Kanagawa, Japan; d Department of Microbiology and Immunology, Keio University School of Medicine, Tokyo, Japan

## Abstract

We report here an HIV-1 recombinant composed of CRF01_AE and subtype B, with a total of eight recombination breakpoints in the *gag-pol* and *vpr-tat* regions. This recombinant was identified from a Myanmarese heterosexual male in Japan and showed the chimera structure identical to recently reported CRF69_01B, detected primarily among men who have sex with men in Japan.

## GENOME ANNOUNCEMENT

A high level of genetic diversity is a hallmark of human immunodeficiency virus type 1 (HIV-1) (1). To date, HIV-1 is classified into four phylogenetic groups: M, O, N, and P (2, 3). Group M, responsible for the vast majority of HIV infections in the world, is further classified into 11 subtypes and sub-subtypes (A1, A2, B, C, D, F1, F2, G, H, K, and J) and more than 70 circulating recombinant forms (CRFs) with various types of unique recombinant forms (http://www.hiv.lanl.gov).

The wide circulation and dual infection of CRF01_AE and subtype B of western origin, as well as Bʹ of Thai origin (4, 5), have played important roles in the emergence of diverse intergenotype recombinants in Japan. To date, a total of four CRFs, including CRF51_01B from Singapore (6), CRF55_01B (7) and CRF59_01B (8, 9) from China, and CRF69_01B recently reported from Japan (10), are known to be composed of CRF01_AE and subtype B of western origin and were found primarily among men who have sex with men (MSM). Here, we describe a genome sequence of a recombinant strain (05JP.MY.CS113SP420) composed of CRF01_AE and subtype B of western origin that was identified in a male Myanmarese resident in Japan. We found that 05JP.MY.CS113SP420 showed the chimera structure apparently identical to that of CRF69_01B, which was recently reported from Japan (10).

The HIV-1 strain was isolated from a consenting 24-year-old Myanmarese heterosexual male in November 2005 in Tokyo by cocultivation with activated peripheral mononuclear cells from a healthy donor (11–13). A near-full-length genome (NFLG) sequence was cloned from proviral DNA from virus culture and was determined as previously described (14–16), using the ABI PRISM 3730XL DNA Analyzer (Applied Biosystems). The NLFG sequence was then subjected to phylogenetic (17) and recombination breakpoint (Simplot) analyses (18).

The NFLG sequence of 05JP.MY.CS113SP420 was 9,112 nucleotides (nt) in size (HXB2: 634 to 9690 nt), spanning the noncoding region (NCR), the *gag*, *pol*, *env*, *tat*, *rev*, *vif*, *vpr*, *vpu*, and *nef* genes, and a 3′ part of the long terminal repeat (LTR). Bootscanning plot and informative site analyses (18) identified a total of eight recombination breakpoints between CRF01_AE and subtype B at the nucleotide positions 1185 ± 15, 1656 ± 3, 1697 ± 13, 4315 ± 5, 4481 ± 15, 4957 ± 31, 5769 ± 20 and 5922 ± 16 (relative to the HXB2 genome). Subregion tree analyses further confirmed the parental origin of each region of the recombinant genome as follows: region I (HXB2: nucleotide positions 790 to 1170), CRF01_AE; region II (nucleotide positions 1200 to 1653), B; region III (nucleotide positions 1659 to 1684), CRF01_AE; region IV (nucleotide positions 1710 to 4310), subtype B; region V (nucleotide positions 4320 to 4466), CRF01_AE; region VI (nucleotide positions 4496 to 4925), subtype B; region VII (nucleotide positions 4988 to 5748), CRF01_AE; region VIII (nucleotide positions 5789 to 5905), subtype B; and region IX (nucleotide positions 5938 to 9690), CRF01_AE. Subregion tree analyses also revealed that the subtype B regions were of western origin, and not the subtype Bʹ lineage (4, 5) associated with blood-borne epidemics in Asia (19, 20). The CRF01_AE regions belonged to Thailand CRF01_AE radiation.

We noted that a chimera structure of 05JP.MY.CS113SP420 appeared to be identical to CRF69_01B, which was recently reported from Japan (10). While CRF69_01B is found primarily among MSM in the Japanese male population (10), it also disseminates into the non-Japanese population at risk of heterosexual exposure.

### Nucleotide sequence accession number. 

The sequence is available in GenBank under the accession number LC027100.
